# Ultrafast exciton energy transfer dynamics in the cryptophyte light-harvesting antenna phycoerythrin 566

**DOI:** 10.3389/fpls.2025.1682154

**Published:** 2025-12-02

**Authors:** Na Guo, Zidong Liang, Xinyu Guo, Zhencheng Huang, Lei Zhang, Mingyuan Xie, Yang Pu, Wenjun Li, Min Chen, Song Qin, Fuli Zhao

**Affiliations:** 1 School of Physics, State Key Laboratory of Optoelectronic Materials and Technologies, Sun Yat-sen University, Guangzhou, China; 2 School of Fisheries, Ludong University, Yantai, China; 3 Bio-Nanotechnology Research Institute, Ludong University, Yantai, China; 4 Yantai Institute of Coastal Zone Research, Chinese Academy of Sciences, Yantai, China; 5 Institute of Life Science, Yantai University, Yantai, China

**Keywords:** photosynthesis, cryptophytes, phycoerythrin, excitation energy transfer, transientabsorption spectroscopy, coherent modified Redfield theory

## Abstract

**Introduction:**

Cryptophytes obtain energy through photosynthetic pigments and transfer it to the photosynthetic center on an ultrafast timescale. The mechanisms of such ultrafast excitation energy transfer (EET) in light-trapping complexes are a major focus of algal research. The closed-form phycoerythrin 566 (PE566) binds chemically distinct chromophores, exhibiting spectral properties that differ from those of other closed-form phycobiliproteins in cryptophytes. Elucidating the ultrafast energy transfer pathway of PE566 may provide a deeper understanding of the mechanisms involved in the initial stages of photosynthesis.

**Method:**

We present a comprehensive description of the ultrafast energy transfer kinetics of PE566 under physiological conditions. A combined approach using ultrafast transient absorption (TA) spectroscopy and coherent modified Redfield theory (CMRT) was employed for theoretical modeling to investigate the ultrafast energy transfer between pigment molecules, including the exciton dynamics in PE566.

**Results and Discussion:**

The results indicate that, in the PE566 dimer, the two phycoerythrobilins (PEBs) possess the highest excitation energies and act as the primary donors in the EET process. The two double-linked bilin584s serve as secondary energy transfer acceptors, exhibiting strong electronic coupling that leads to coherent delocalization of excited states. Two single-linked bilin584s and two bilin618s constitute the four lowest-energy exciton states. Ultimately, two efficient EET pathways were identified, with the lowest-energy bilin618s serving as the terminal acceptors for energy transfer in PE566. Our work clarifies the internal EET mechanism of *Cryptophyta* PE566, which may advance photophysical studies of phycobiliprotein systems.

## 1 Introduction

**Figure 1:**
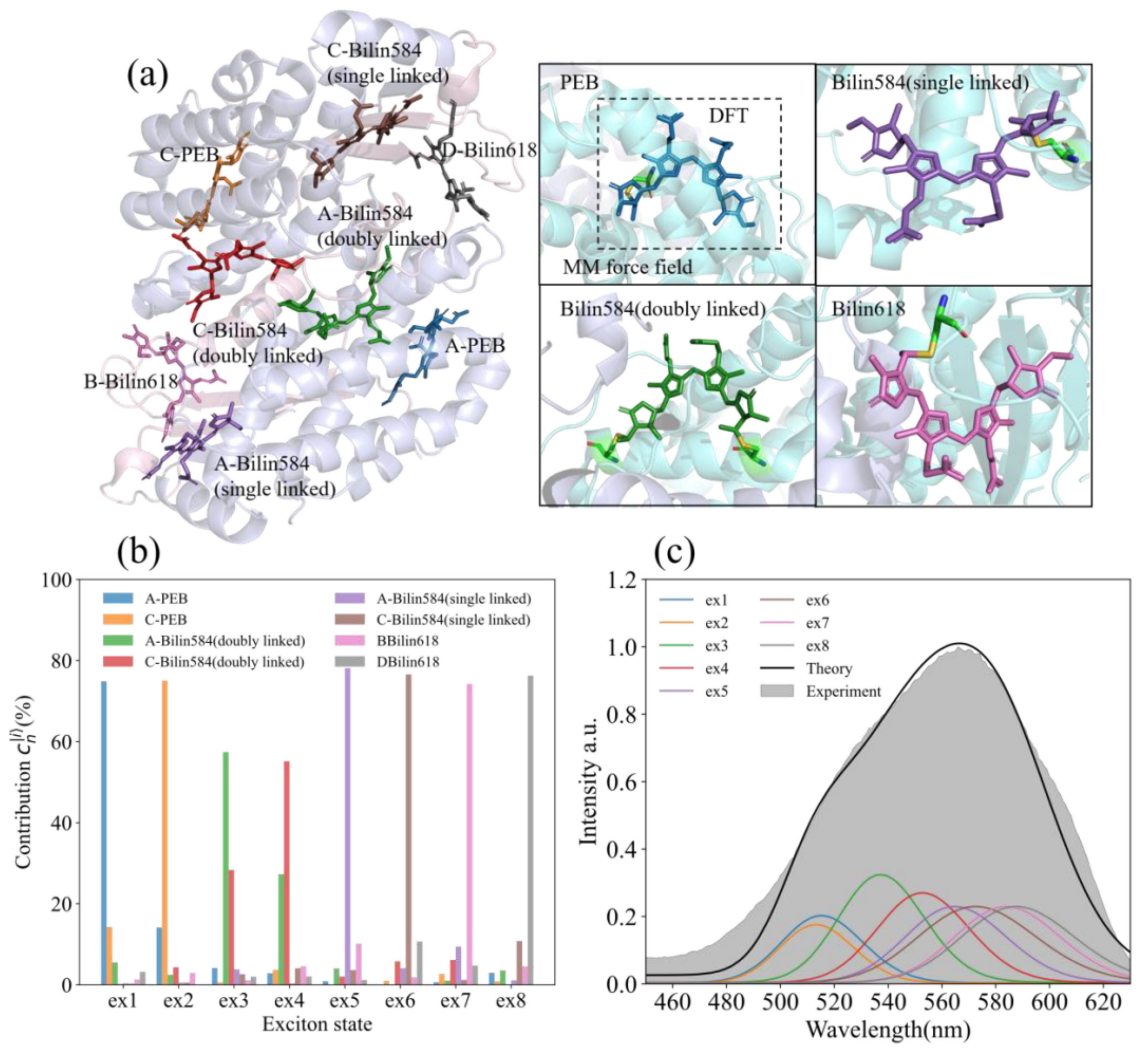
The protein structure, steady-state absorption spectrum, and exciton state of PE566. **(a)** The protein structure and chromophore of PE566 (for the PDB entry 7T8S). Molecular structures reveal the origin of spectral variation in cryptophyte light-harvesting antenna proteins (Michie et al., 2023). The four subfigures on the right side show the four kinds of chromophores contained in PE566 (PEB: bilin584, doubly linked; bilin584, single linked; and bilin618). Our quantum chemical calculations consist of two layers: a high-precision density functional theory (DFT) calculation that describes the chromophores and the amino acids they are linked to, contained within the dotted lines, and the remaining protein environment described by molecular mechanics (MM) force fields. **(b)** Expansion coefficient of the PE566 eigenstate to the chromophore excited state. **(c)** Theoretical absorption spectrum of PE566 (black solid line) and experimental results (gray fill); the theoretical absorption spectrum is decomposed into eight exciton superpositions (color lines).

Photosynthesis is the core process of energy conversion in the biosphere, in which light-trapping antennae proteins efficiently transfer photoexcitation energy to the reaction center through a precisely assembled pigment network, with energy transfer lifetimes reaching the femtosecond to picosecond scale (Zehr and Kudela, 2009; Eaton-Rye et al., 2012; Scholes et al., 2012; Thyrhaug et al., 2018). This ultrafast kinetic feature illustrates the physical principles underlying light quantum utilization by photosynthetic organisms and provides important insights for the development of artificial light energy devices. Consequently, the investigation of pigment excitation kinetics has emerged as a major focus in elucidating the mechanisms of algal photosynthesis (Turner et al., 2012). The classical Förster resonance energy transfer theory describes dipole–dipole interactions in weakly coupled systems. Recent studies have shown that quantum coherence effects markedly enhance the excitation energy transfer (EET) efficiency of specific pigment–protein complexes (Dean et al., 2016; Duan et al., 2017, 2022). As a unique branch of photosynthetic organisms, cryptophytes possess light-trapping systems composed of phycobiliproteins (PBP), in which heterodimers aggregate in the cyst-like lumen to form a highly efficient light-energy-trapping network (Merritt and Richardson, 2024). Typical representatives include phycocyanin 645 (PC645) (*Chroomonas* sp.) and phycoerythrin 545 (PE545) (*Rhodomonas* sp.), whose quantum coherence in light-trapping systems has been investigated using time-resolved spectroscopy (Collini et al., 2010). These studies provide a key basis for understanding the evolutionary advantages of cryptophyte photosystems. Phycoerythrin 566 (PE566; cryptophyte *Cryptomonas pyrenoidifera*) is an important member of the cryptophyte family, belonging to the (αLβ)·(αLβ) heterodimer in closed form. PE566 contains eight light-trapping pigments, with bilin618 bound to the α-subunit, classified as B-bilin618 and D-bilin618 depending on the chain. The β-subunit is double-linked to A-bilin584 and C-bilin584 at β50/61. β158 is single-linked to A-bilin584 and C-bilin584. The β82 sites on the A and C chains are linked to PEBs, designated as A-PEB and C-PEB (**Figure 1a**). Recent investigations of crystal structure have demonstrated that PE566 exhibits a similar structure to the closed form of PE545. Notably, chemical modification of bilin618 results in planarization of its conjugation system, causing a red shift in the absorption spectrum to 566 nm. This structural specificity facilitates studies of the dynamic re; relationship between cryptophyte protein structure and function (MacColl et al., 1990; Michie et al., 2023). Recent research on the closed conformation of cryptophytes has revealed strongly coupled coherent EET associated with this structural feature. Accordingly, we focus on the structural and energy transfer properties of PE566 in its closed conformation.

In this study, we aim to investigate the detailed EET process in the PE566 dimer, starting from a general exciton model and integrating experimental results from ultrafast laser spectroscopy. Utilizing the quantum mechanical/molecular mechanics (QM/MM)-Own N-layered Integrated molecular Orbital and molecular Mechanics (ONIOM) combined with the coherent modified Redfield theory (CMRT) method, we calculated the EET process in the excited state of PE566. The global excited-state energy level distribution was constructed using the Frenkel exciton model, and the kinetic features of the excited state were quantitatively analyzed based on CMRT. Moreover, the pigments were selectively excited using transient absorption (TA) ultrafast spectroscopy measurements, and the energy transfer kinetics of the eight pigments were analyzed through global fitting. The complete energy transfer pathway among the PE566 pigments was thoroughly analyzed.

## 2 Materials and methods

### 2.1 Protein extraction and purification

In this paper, the cryptophyte *Cryptomonas pyrenoidifera* was cultured at 20°C in BG-11 medium supplemented with vitamins B1, B6, and B12. The algae biomass was separated from the culture medium by centrifugation (4,000 rpm, 10 min, 4 °C) and stored at − 80 °C for subsequent purification. At the start of the purification process, 0.05 mol/L phosphate buffer (pH 7.4) was added to the thawed algal cells at a mass-to-volume ratio of 1:10. After three freeze–thaw cycles, centrifugation (12,000 rpm, 30 min, 4°C) was performed to obtain a supernatant containing PE566. The protein was precipitated first with 30% saturated ammonium sulfate and then with 90% saturated ammonium sulfate, followed by centrifugation (12,000 rpm, 20 min, 4°C) to obtain the precipitate containing PE566. The precipitate was dissolved in a small amount of 0.05 mol/L phosphate buffer. The resulting PE566 solution was passed through a 0.22-μm filter and further purified by FPLC using an a butyl-S agarose 6 Fast Flow hydrophobic interaction chromatography column (GE Healthcare, Chicago, Illinois, USA), with real-time monitoring at 280 nm.

Briefly, to equilibrate the column, five column volumes of equilibration buffer (0.05 mol/L phosphate buffer containing 1.7 M (NH_4_)_2_SO_4_) were pumped through the medium at a flow rate of 2 mL/min. The column was then washed with 0.05 mol/L phosphate buffer containing 1.2 M (NH_4_)_2_SO_4_ at the same flow rate to ensure complete elution of impurities. Finally, analytical-grade PE566 protein (A566/A280 = 5.70) was eluted with 0.05 mol/L phosphate buffer, immediately frozen in liquid nitrogen, and stored at − 80°C.

### 2.2 Spectroscopy device

The experimental setup for femtosecond transient absorption is shown in **Supplementary Figure S1**. The setup employed a regenerative Ti:sapphire amplifier system (Astrella, Coherent, Santa Clara, California, USA; 35 fs, 800 nm) producing an output pulse at a center wavelength of 800 nm, with a repetition frequency of 1 kHz, pulse width of 35 fs, and pulse power of approximately 7 W. The output beam was split into two using a beam splitter. One of the pulses was passed through an optical parametric amplifier (OPerA-Solo, Light Conversion, Vilnius, Lithuania) to tune the center wavelength as the pump beam, and a mechanical chopper was then used to adjust the pump repetition rate to half that of the detector beam. The other pulse served as the probe beam and was directed into the delay line (M-ILS200HA, Newport, Irvine, California, USA). The TA experiment covered a time range from 20 fs to 1 ns to control the delay between the pump and probe beams. After passing through the translation stage, the pulse was focused on heavy water to generate supercontinuum white light as the probe beam. A 750-nm short-wavelength pass filter and a green glass sheet were used to remove the 800-nm fundamental frequency laser component from the probe light before reaching the sample, preventing detection of transient absorption signals above 750 nm. A fiber spectrometer (Ocean Optics, Orlando, Florida, USA) was used to record the probe white light in external trigger mode. The angle between pump and probe beams was set to the magic angle (54.7°). The optical path of the sample was 5 mm. A small magnetic needle was used to stir the sample to prevent light-induced damage.

All the spectral data obtained in the experiment were fitted for analysis using Glotaran (Snellenburg et al., 2012). Global analysis is an algorithm that fits the decay process at different wavelengths through multiple exponentials. By modeling the continuous decay, the result outputs evolution-associated spectra (EAS) and decay lifetimes. The shortest lifetime that can be resolved, however, depends on the temporal resolution of the experiment.

Steady-state photoluminescence (PL) and time-resolved photoluminescence (TRPL) measurements were performed using an FLS1000 Fluorescence Spectrometer (Edinburgh Instruments, Livingston, United Kingdom). For the TPL measurements, the excitation source was a supercontinuum white-light laser operating at 20 MHz.

### 2.3 Theoretical model and computational methods

In this study, we employed the Frenkel exciton model to describe the global electron excitation of PE566. Within the framework of this model, the system Hamiltonian consists of the following components (Valkunas et al., 2013).

(1) $$\begin{array}{c} H=H^{el}+H^{Coul}+H^{ph}+H^{el-ph}+H^{reorg} \end{array}$$

Here


$$H^{el}=\sum_{n=1}^{N}E_{n0}|n\rangle \langle n|$$

$$H^{Coul}=\sum_{n\neq m}V_{nm}|n\rangle \langle m|$$

$$H^{ph}=\sum_{n=1}^{N}\sum_{i}\left(\frac{p_{ni}^{2}}{2M_{ni}}+\frac{1}{2}M_{ni}\omega_{ni}^{2}q_{ni}^{2}\right)$$

$$H^{el-ph}=\sum_{n=1}^{N}u_{n}|n\rangle \langle n|,u_{n}=\sum_{i}\left(M_{ni}\omega_{ni}^{2}q_{ni}d_{ni}\right)$$

$$H^{reorg}=\sum_{n=1}^{N}\lambda_{n}|n\rangle \langle n|,\lambda_{n}=\sum_{i}\left(\frac{1}{2}M_{ni}\omega_{ni}^{2}d_{ni}^{2}\right)$$

where |n〉 represents the n-th chromophores. In PE566 with eight chromophores, N=8. $E_{n0}$ is the electronic excitation energy of the *n*th chromophores. $V_{nm}$ is the Coulombic coupling between the *n*th and *m*th chromophores. Each chromophore is coupled to its own photonic bath, characterized by its specific momentum $p_{ni}$ and displacement $q_{ni}$ of the *i*th phonon mode. $H^{el-ph}$ represents the electron–phonon coupling of the system. This is caused by the difference in the equilibrium positions of the potential energy surfaces between the excited state and the ground state. $u_{n}$ donates the electron–phonon coupling energy of the *n*th chromophores. $\lambda_{n}$ is the reorganization energy of the *n*th chromophores, and it represents the excited-state relaxation energy.

To determine the Hamiltonian of PE566 in Equation 1, a two-layer QM/MM-ONIOM model (Vreven and Morokuma, 2006) was employed to calculate: (i) the electron excited-state energy and electron density distribution, (ii) the electron coupling energy between chromophores, and (iii) the reorganization energy of each chromophore, which reflects the interaction between the chromophore and its environmental bath. The high layer contains the chromophore and its protein-linked cysteine (for doubly linked chromophores, both linked cysteines are included) to accurately describe the electron density distribution of the chromophore. Density functional theory was used to calculate the high layer with high precision, using the hybrid functional b3lyp (Stephens et al., 1994) in combination with the Pople double-zeta basis set, 6-31G(d) (Petersson et al., 1988). The low layer includes all remaining atoms in the PE566 protein and was treated using the UFF molecular force field (Rappe et al., 1992) to simulate the protein environment. Electronic excited states were calculated using time-dependent density functional theory (TD-DFT) based on fully optimized ground-state geometries at the same level of theory. In addition, the S1 excited state of the PE566 ONIOM model was geometrically optimized to calculate the reorganization energy of each chromophore within the protein environment. All calculations were performed using the Gaussian 16 software package (Hehre et al., 1972).

In this study, the electron coupling energy between PE566 chromophores was calculated using transition charges from the electrostatic potential (TrEsp) method (Madjet et al., 2006; Renger, 2009), which is expressed as Equation 2:

(2) $$\begin{array}{c} V_{DA}=\sum_{i\in D,j\in A}\frac{q_{i}q_{j}}{\left|R_{ij}\right|} \end{array}$$

For the system described in Equation 1, the evolution of the density matrix ρ of the entire system follows von Neumann equation (here, we use the simplification ℏ=1) as Equation 3:

(3) $$\begin{array}{c} \dot{\rho }(t)=-i[H,\rho (t)] \end{array}$$

We follow the framework of modified Redfield theory (Zhang et al., 1998; Yang and Fleming, 2002) and apply an exciton basis {|k〉}, decomposing the Hamiltonian into two parts—the reference and the perturbation—via Equation 4:

(4) $$\begin{array}{c} H=H_{0}+H^{\prime } \end{array}$$

here $H_{0}=\sum_{k}|k\rangle \left(H^{e}+H^{el-ph}\right)\langle k|+H^{ph}$ represents the eigenmatrix of the electronic Hamiltonian of the system. |k〉 is the exciton state, which represents a delocalized linear combination of site excitations. In PE566 with eight chromophores, it can be expressed as $|k\rangle =\sum_{\text{n}}^{8}c_{n}^{k}|n\rangle$. $H^{\prime }$ is the perturbation part, representing the electron–phonon coupling between exciton states, expressed as $H^{\prime }=\sum_{k.k^{\prime }\left(k\neq k^{\prime }\right)}|k\rangle H^{el-ph}\langle k^{\prime }|$. Combining with Equation 1, we obtain Equation 5:

(5) $$\begin{array}{c} H_{kk}=\sum_{n}\{c_{n}^{k}c_{n}^{k}*(E_{n0}+u_{n})+{\left(c_{n}^{k}c_{n}^{k}*\right)}^{2}\lambda_{n}+E_{n}^{ph}\}+\sum_{n\neq m}c_{n}^{k}c_{m}^{k}*V_{nm}\\ \begin{array}{c} H_{kk^{\prime }}^{'}=\sum_{n}c_{n}^{k}c_{n}^{k}*u_{n} \end{array} \end{array}$$

The system population transfer rate in the exciton basis is described using modified Redfield theory based on perturbation theory (Yang and Fleming, 2002; Silbey, 1996) (detailed derivation is shown in the **Supplementary Material**), via Equation 6:

(6) $$\begin{array}{c} K_{kk^{\prime }}(t)=2Re\int_{0}^{t}d\tau F_{k^{\prime }}^{*}(\tau )A_{k}(\tau )N_{kk^{\prime }}(\tau ) \end{array}$$

with F(t) and A(t) are the emission and absorption spectra of the exciton state, respectively, which are expressed as Equation 7:

(7) $$\begin{array}{c} F_{k}(t)=\exp\{-i(E_{k0}-\lambda_{kkkk})t-g_{kkkk}^{*}(t)\}\\ A_{k}(t)=\exp\{-i(E_{k0}+\lambda_{kkkk})t-g_{kkkk}(t)\} \end{array}$$

here $E_{k0}=\sum_{n}\{c_{n}^{k}c_{n}^{k}*E_{n0}\}$,

(8) $$\begin{array}{c} \lambda_{kk^{\prime }k^{\prime \prime }k^{\prime \prime \prime }}=\sum_{n}c_{n}^{k}c_{n}^{k^{\prime }}c_{n}^{k^{\prime \prime }}c_{n}^{k^{\prime \prime \prime }}\lambda_{n}\\ g_{kk^{\prime }k^{\prime \prime }k^{\prime \prime \prime }}=\sum_{n}c_{n}^{k}c_{n}^{k^{\prime }}c_{n}^{k^{\prime \prime }}c_{n}^{k^{\prime \prime \prime }}g_{n}\;,\\ g_{n}(t)=\int_{0}^{t}dt_{1}\int_{0}^{t_{1}}dt_{2}\text{Tr}\{u_{n}(t_{1})u_{n}(t_{2})\rho_{n}^{ph}\} \end{array}$$

and

(9) $$\begin{array}{c} N_{kk'}(\tau )=\exp\{2g_{kkk'k'}(\tau )+2i\lambda_{kkk'k'}\tau \}\\ \times \{{\ddot{g}}_{k'kkk'}(\tau )-({\dot{g}}_{kkkk'}(\tau )-{\dot{g}}_{k'k'kk'}(\tau )-2i\lambda_{k'k'kk'})\times ({\dot{g}}_{k'kkk}(\tau )-{\dot{g}}_{k'k'k'k}(\tau )-2i\lambda_{k'k'k'k})\} \end{array}$$

In addition, similar to the light-harvesting complexes PC645 and PE545 previously reported (Collini et al., 2010; Lee et al., 2017; Blau et al., 2018; Cui et al., 2020), PE566 contains eight chromophores, and the two doubly linked Bilin584 probably form a dimer structure with obvious coherence. Therefore, it is necessary to consider the coherence between PE566 chromophores. We referred to the CMRT model proposed by Ai et al. (2013); Chang and Cheng (2015), and Hwang-Fu et al. (2015) and incorporated corrections for coherent states based on Equation 9. The CMRT approach uses a non-Markovian secular quantum master equation to describe the time evolution of the reduced density matrix (RDM) in the exciton basis. Corrections for coherent states (nondiagonal elements of the RDM) can be expressed as Equation 10:

(10) $$\begin{array}{c} {\dot{\sigma }}_{kk^{\prime }}(k\neq k^{\prime })=\{-i(E_{k0}-E_{k^{\prime }0})-[R_{kk^{\prime }}^{pd}(t)+\frac{1}{2}\sum_{k^{\prime \prime }\neq k,k^{\prime }}(K_{k^{\prime \prime }k}(t)+K_{k^{\prime \prime }k^{\prime }}(t))]\}\sigma_{kk^{\prime }} \end{array}$$

The first term in Equation 10 accounts for the coherent oscillation between exciton states, while the second term represents the dephasing process arising from the zero-order Hamiltonian. The dephasing dynamics in the CMRT framework are expressed as Equation 11.

(11) $$\begin{array}{c} R_{kk^{\prime }}^{pd}(t)=Re({\dot{g}}_{kkkk}(t)+{\dot{g}}_{k^{\prime }k^{\prime }k^{\prime }k^{\prime }}(t)-2{\dot{g}}_{kkk^{\prime }k^{\prime }}(t)) \end{array}$$

By combining Equation 10 with Equation 6, we derive the mathematical description of the EET dynamics of the RDM within the CMRT framework, expressed as Equation 12:

(12) $$\begin{array}{c} {\dot{\sigma }}_{kk'}=\delta_{kk'}\sum_{k''\neq k}\left[K_{kk''}(t)\sigma_{k''k''}(t)-K_{k''k}\sigma_{kk}(t)\right]\\ \{-i(E_{k0}-E_{k;0})-[R_{kk'}^{pd}(t)+\frac{1}{2}\sum_{k''\neq k,k'}\left(K_{k''k}(t)+K_{k''k'}(t)\right)]\}\sigma_{kk'} \end{array}$$

In our numerical calculations, the line shape function represented by Equation 8 describes the coupling of the system with the phonon bath, which is characterized using spectral density. The spectral densities, J(ω), reflect the coupling intensity between chromophore and environmental bath, expressed as ${\text{J}}_{\text{a}}(\text{ω})=\text{π}/2\sum_{\text{i}}^{\text{N}(\text{a})}({\text{c}}_{\text{i}}/{\text{ω}}_{\text{i}})\text{δ}(\text{ω}-{\text{ω}}_{\text{i}})$. In this paper, the Drude–Lorentz function ${\text{J}}_{\text{n}}(\text{ω})=2{\text{λ}}_{\text{n}}\text{ωγ}/(1+{\left(\text{ωγ}\right)}^{2})$ (Mirkovic et al., 2007; Huo and Coker, 2011; Wu et al., 2012; Cui et al., 2020) is used to construct chromophore spectral density. The reorganization energy ${\text{λ}}_{\text{n}}$ reflects the Stokes shift caused by relaxation of the system and its environment. We used the lowest absorption peak measured experimentally and the highest emission peak from the fluorescence emission spectrum, combined with TD-DFT calculations, to determine the recombination energy size of each chromophore. In our calculations, we set γ to $1{\text{ps}}^{-1}$ to simulate relaxation of the system within the environment bath. The spectral density parameters of different chromophores in PE566 are presented in the **Appendix**.

The theoretical absorption and fluorescence emission spectra of PE566 are decomposed into eight exciton states, with the spectral line shape of each exciton state determined by its spectral density, reorganization energy, and exciton state energy (Fleming and Cho, 1996; Loco and Cupellini, 2019), as specifically expressed in Equation 7. We employ the harmonic oscillator approximation, via Equation 13.

(13) $$\begin{array}{c} g_{nnnn}(t)=-\int_{0}^{\infty }d\omega \frac{J_{nnnn}(\omega )}{\omega^{2}}\times [\coth(\frac{\hbar \omega }{2kT})(\cos(\omega t)-1)-i(\sin(\omega t)-\omega t)] \end{array}$$

Due to the delocalization of the exciton state, the reorganization energy and spectral density function of each exciton state account for the contributions of all chromophores to that exciton state (Novoderezhkin and Van Grondelle, 2010). Therefore, we can obtain Equation 14:

(14) $$\begin{array}{c} J_{kk^{\prime }k^{\prime \prime }k^{\prime \prime \prime }}(\omega )=\frac{1}{2\pi }\int_{-\infty }^{\infty }d\omega \sum_{n}c_{n}^{k}c_{n}^{k^{\prime }}c_{n}^{k^{\prime \prime }}c_{n}^{k^{\prime \prime \prime }}J_{n}(\omega ) \end{array}$$

## 3 Results and discussion

### 3.1 Computational model

The crystal structure of PE566 used in our computational model is shown in **Figure 1a** (PDB ID: 7t8s). PE566 contains four types (illustrated in the subfigure on the right side of **Figure 1a**), totaling eight chromophores. These eight chromophores primarily contribute to the system’s excitation and participate in the EET dynamics. We employed the hierarchical QM/MM-ONIOM method to calculate the excited-state properties and EET processes of these chromophores within the PE566 protein environment. The specific computational methodology is described in Section 2.2.

We decompose the absorption spectra of PE566 as a linear superposition of the absorption line shapes of its eigenstates. Each eigenstate is further expressed as a linear combination of chromophore local excitations. The superposition coefficients are determined by the system Hamiltonian (the full Hamiltonian of PE566 is in **Appendix 1**), where the off-diagonal elements represent the electron coupling potential between chromophores. In this study, the electron coupling energies of PE566 chromophores are calculated using the TrEsp method (Madjet et al., 2006; Renger, 2009) implemented in the Multiwfn package (Lu and Chen, 2012).

The expansion coefficients of each exciton state of the chromophores are shown in **Figure 1b**. Exciton states 1 (ex1) and 2 (ex2), which have the highest energies, are primarily contributed by A-PEB and C-PEB. The energy gap between these two PEBs and the other six chromophores is an order of magnitude larger than the electron coupling energy. Consequently, the remaining six chromophores contribute minimally to these two exciton states. Furthermore, ex1 and ex2 can be classified as local excitations because A-PEB and C-PEB exhibit very low electronic coupling energy (~ 5.5 ${\text{cm}}^{-1}$), which is insufficient to produce significant delocalization between the two PEBs.

Exciton states 3 (ex3) and 4 (ex4) are composed of the two double-linked bilin584s located at the center of PE566. These chromophores have two main-chain protein-binding sites, which stabilize their geometry and place them at a higher potential energy than the single-linked bilin584. Similar to previously reported light-harvesting complexes (Collini et al., 2010; Lee et al., 2017; Blau et al., 2018; Cui et al., 2020), the two double-linked bilin584s also exhibit strong electron coupling (~ 200.3 ${\text{cm}}^{-1}$) in our calculations. Therefore, ex3 and ex4 represent delocalized exciton states consisting of these two double-linked bilin584s. Owing to the influence of electron coupling, ex3 and ex4 exhibit an energy level splitting of approximately 550 ${\text{cm}}^{-1}$, which is roughly twice the energy difference between the two individual site states.

The two single-linked bilin584s, with an absorption peak at approximately 575 nm, form ex5 and ex6. Similarly, the two bilin618s, which have an absorption peak around 590 nm, mainly contribute to energy transfer to the terminal ex7 and ex8. In addition, based on the Hamiltonian and the spectral line shape in Equation (A5), we calculated the absorption spectrum of PE566 as a linear superposition of exciton state absorption spectra. The calculated results were compared with the steady-state absorption spectra of PE566 measured experimentally (**Figure 1b**). In the shorter-wavelength region, the calculated absorption intensity is lower than the experimentally measured values. This discrepancy may be due to high-frequency vibrational modes coupled to the bath that were ignored in our calculation, resulting in an overly narrow exciton state absorption line shape. Even so, near the absorption peak of PE566, the theoretical calculations are in good agreement with the experimental results.

**Figure 2:**
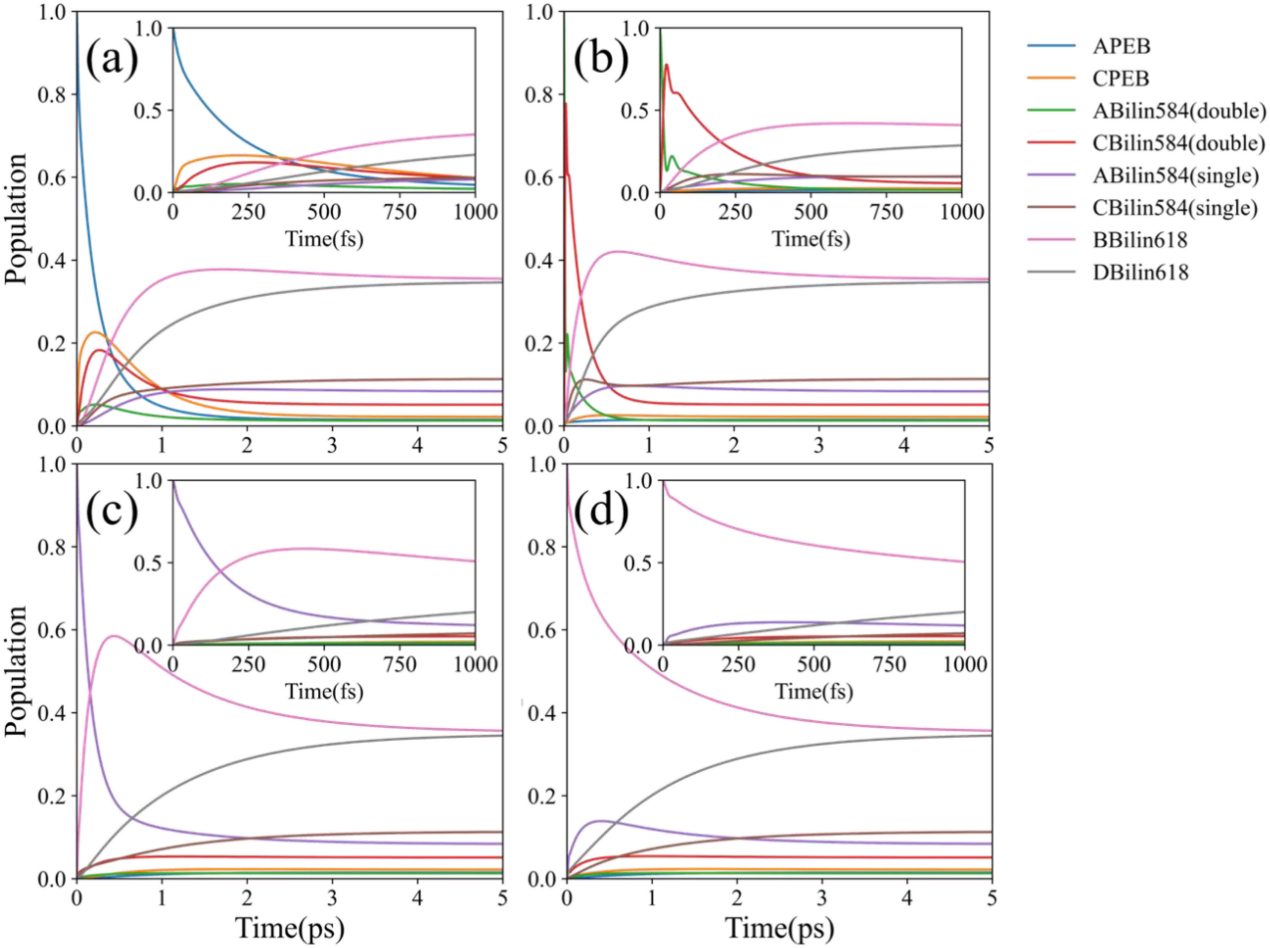
EET dynamics simulation for different initial states at room temperature (RT) (*T* = 300 K). For each initial condition, both long- and short-term dynamic simulations were conducted (subgraphs in each graph). **(a)** Population evolution at the initial excitation state σ(0) on A-PEB. **(b)** Population evolution at the initial excitation state σ(0) on double-linked C-bilin584. **(c)** Population evolution at the initial excitation state σ(0) on single-linked A-bilin584. **(d)** Population evolution at the initial excitation state σ(0) on B-bilin618.

The dynamic simulation shows the EET energy flow process of PE566 under different initial excitations at *T* = 300 K (the same temperature as in the experiments). To simplify the model, a single excitation approximation was used in the numerical simulation, with the initial state σ(0) set on a chromophore site state. The simulation was run over a 5-ps timescale to capture the complete process of energy transfer between the chromophores’ excited states. Additionally, ultrafast EET dynamics within a 1-ps timescale are presented in the subgraphs of each figure to highlight population evolution between excited states over short times. **Figure 2a** shows the energy flow within the PE566 when the initial excitation is on A-PEB, which has the highest site energy. The energy mainly flows from A-PEB to the double-linked bilin584 dimer within about 0.5 ps, followed by transfer to lower-energy states, eventually reaching a steady state. Meanwhile, the electron coupling potential between A-PEB and C-PEB is approximately 5.5 ${\text{cm}}^{-1}$, which is too weak to form a delocalized excitation. Therefore, no coherent oscillations were observed between the two PEBs.

**Figure 2b** shows the energy flow when the initial excitation is on the double-linked C-bilin584. The double-linked C-bilin584 and double-linked A-bilin584 are located in the center of PE566 and undergo strong electron coupling. The energy flow between the two double-linked bilin584s occurs as a fast coherent process, completed within 200 fs. In our calculations, although the two double-linked bilin584s have strong coherent coupling (200.3 ${\text{cm}}^{-1}$) and are close enough to generate quantum beats, no long-term coherent oscillations were observed between them. Only a few coherent oscillation periods appear in the ultrafast EET dynamics simulation. This is because the site energy gap between the two double-linked bilin584 is relatively large (320.2 ${\text{cm}}^{-1}$), which reduces delocalization and leads to rapid coherent dephasing.

Moreover, A-bilin584 and B-bilin618, as well as C-bilin584 and D-bilin618, respectively, form two sets of fast transfer energy pathways, as shown in **Figures 2c, d**. The two bilin618s with the lowest energy in PE566, B-bilin618 and D-bilin618, act as the terminal energy acceptors. When these lower-energy exciton states are initially excited, the populations of the two PEBs and the bilin584 dimer with higher exciton energy hardly increase. In this case, the high energy gap prevents significant population transfer toward the two PEBs and the bilin584 dimer. This observation is consistent with the results obtained from the transient absorption spectroscopy experiments, which will be discussed in Section 2.3.

Using the QM/MM-ONIOM method and CMRT, we numerically calculated the EET process of the PE566 exciton states. Our calculation results indicate that the PEBs have the highest excitation energies and act as the initial energy donors in the EET process. The two doubly linked bilin584s at the center of PE566 have lower energies and serve as secondary energy transfer receptors. Strong electron coupling between them forms a coherent, delocalized excited state, allowing rapid energy transfer. The two single-linked bilin584s and the two bilin618s constitute the four lowest-energy exciton states. Ultrafast EET dynamics simulations clearly identify the energy flow paths starting from the highest-energy PEBs within PE566 (exciton states contract toward the chromophore site states):

1: APEB→Abilin584(doubly linked)→Abilin584(single linked)→Bbilin618
2: CPEB→Cbilin584(doubly linked)→Cbilin584(single linked)→Dbilin618

### 3.2 Transient photoexcitation dynamics

We selected the pigment pump wavelengths for the TA experiment based on the excitation energy calculations (**Figure 1c**). Three different pump excitation wavelengths—533, 566, and 585 nm—were used to excite the sample. From the steady-state spectra analysis, the 533-nm excitation predominantly excites the double-linked A-bilin584, 566 nm mainly excites the double-linked C-bilin584 exciton, and 588 nm corresponds to the single-linked bilin584s. The probe light wavelength ranged from 410 to 700 nm, recording the transient absorption spectra of the PE566 protein at room temperature. We used three to four components in the global analysis fitting with Glotaran, depending on the dataset (Van Stokkum et al., 2004). This method generates EAS and their corresponding time constants through mathematical modeling of the dynamic process. Each EAS follows an exponential decay law, with its intensity gradually decreasing over time and being replaced by subsequent EAS components (**Table 1**).

**Table 1:** Time constants of PE566 obtained from global fitting.

Excitation	τ1	τ2	τ3	τ4	RMS
533 nm	0.69	4.7	29	660	0.48%
566 nm	0.63	3.1	27.4	560	0.57%
585 nm	0.83		14.5	823	0.56%

*τ* is expressed in picoseconds. The relative errors are estimated to be 10% for the lifetimes obtained from TA measurements. The last column indicates the RMS error of the fitting.

#### 3.2.1 Excitation at 533 nm, RT

**Figure 3:**
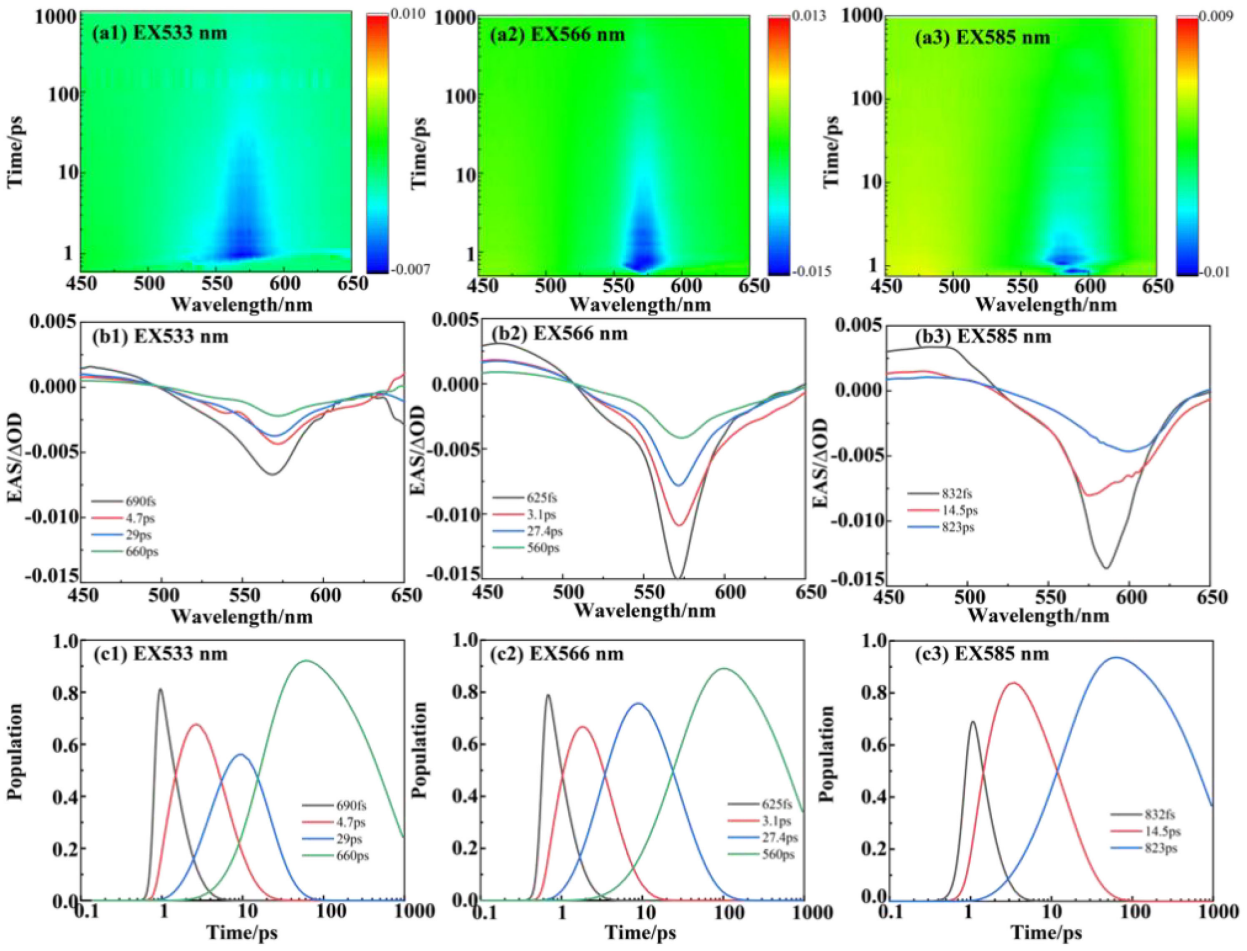
The femtosecond transient spectroscopy of PE566. **(a)** (1–3) Transient absorption contour maps at excitation wavelengths of 533, 566, and 585 nm, respectively. **(b)** (1–3) Evolution-associated spectra (EAS) at excitation wavelengths of 533, 566, and 585 nm, respectively. **(c)** (1–3) Population evolution of the corresponding species at excitation wavelengths of 533, 566, and 585 nm, respectively. The RMS error of the fit is given in **Table 1**.

For the dataset with a 533-nm excitation wavelength, we set up four components and constructed the energy transfer model. **Figures 3a** (1–3) shows the transient absorption contour maps at excitation wavelengths of 533, 566, and 585 nm, respectively. The EAS curve at 533 nm excitation indicates direct excitation of the double-linked A-bilin584. A clear ground-state bleaching process is observed at wavelengths beyond 500 nm, confirming that the PEB chromophore is excited. The peak of the first EAS curve is located at 566 nm. Compared with **Figure 3b** (2), the ground-state bleaching process under 530 nm excitation shows a broader bleaching effect in the 520–550-nm range (corresponding to the absorption region of the double-linked A-584bilin). Therefore, the decay time of approximately 0.69 ps represents the energy transfer from ex1/2 to ex3/4. The second EAS species decays within about 4.7 ps, with a linear peak near 569 nm, corresponding to the excitation of ex3/4. The energy is then transferred from ex3/4 to ex5/6. In the third species process, the absorption peak at 577 nm corresponds to the absorption range of ex5/6. Finally, a relaxation decay process occurs in the terminal pigment molecules. We attribute the final 1.42 ns component to this terminal relaxation. The kinetic signals of the process at 533 nm excitation are illustrated in **Supplementary Figure S3**. The signal at 530 nm corresponds to the ground-state bleaching of the pigment double-linked A-bilin584, exhibiting a rapid decay rate. The signal at 566 nm includes both the ground-state bleaching of the pigment and the excited-state emission. The stronger excited-state emission detected at 588 nm shows a progressively slower decay rate. From these observations, a sequential model of energy transfer can be constructed: PEB→bilin584 (double-linked) (~ 0.69 ps)→C-bilin584 (double-linked) (~ 4.7 ps)→bilin584 (single-linked) (~ 29 ps)→bilin618 (~ 660 ps).

#### 3.2.2 Excitation at 566 nm, RT

The pump excitation at 566 nm corresponds to double-linked C-bilin584 in the steady-state spectra. Compared with the 533-nm excitation, the absorption shoulder at 530 nm is weaker at the 566-nm excitation wavelength (**Figure 3a**(2)). The main absorption peak is concentrated near 568 nm, and the first appearance of the transient species (black line) shows an essentially bleached signal, including the main band at 566 nm. From the EAS curve peaks, it is evident that the bleaching signals in the 520–550-nm range are weaker than those observed under 533 nm excitation. This implies that the main contributor under 566 nm excitation shifts to the pigment single-linked bilin584 corresponding to this band. The sequential model of energy transfer under 566 nm excitation is as follows: PEB→bilin584 (double-linked) (~ 3.1 ps)→bilin584 (single-linked) (~ 27 ps)→bilin618 (~ 560 ps).

#### 3.2.3 Excitation at 585 nm, RT

The ground-state bleaching signal emerges after 525 nm, indicating that the PEBs are not excited. At the pump wavelength of 585 nm (**Figure 3a**(3)), the lower-energy pigment, single-linked bilin584 of PE566, is selectively excited. The first transient species exhibits a ground-state bleaching signal with a peak near 585 nm. This first species (black line) shows a negative signal with a peak at 588 nm, corresponding to an excitation energy transfer of 0.89 ps. The second species (red line) undergoes a redshift, attributed to energy transfer to single-linked bilin584. The final species corresponds to terminal energy relaxation. The sequential model of energy transfer under 585 nm excitation is:

bilin584 (double-linked) (~ 0.89 ps)→bilin584 (single-linked) (~ 14.5 ps)→ bilin618 (~ 823 ps).

**Figure 4:**
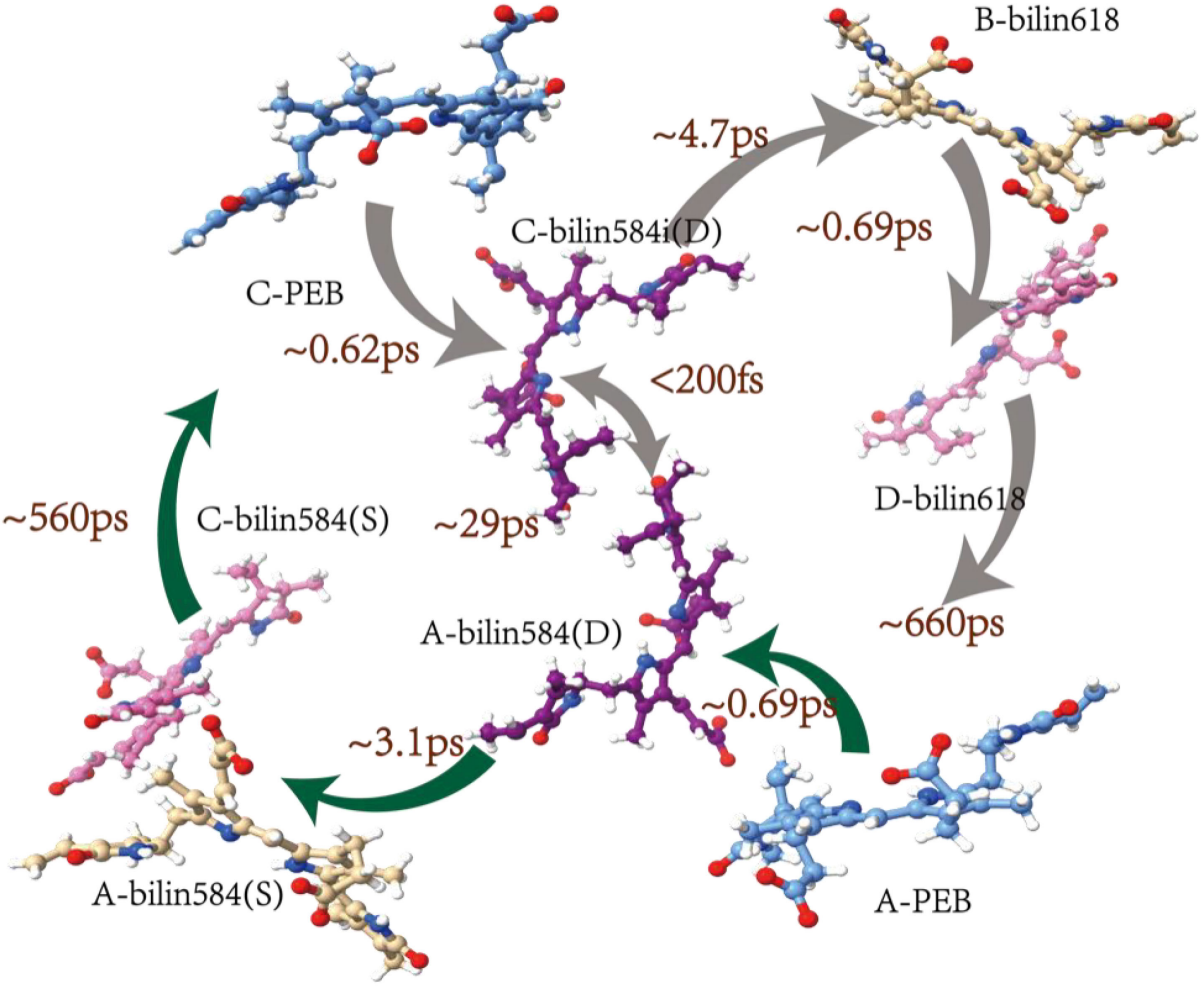
Model for energy transfer in PE566. The energy flow and corresponding lifetimes are indicated on the crystal structure of PE566.

Therefore, the excitation energy transfer kinetics of the PE566 chromophore environment were plotted based on the obtained sequence model (**Figure 4**).

## 4 Conclusions

We investigated the energy transfer mechanism in PE566 by combining ultrafast TA and theoretical calculations. The results show that the excited-state dynamics of PE566 are influenced by its distinctive structural characteristics. Utilizing the QM/MM methodology and CMRT, we elucidated efficient pathways for energy transfer in PE566. The PEB chromophore is regarded as the donor with the maximum excitation energy in the dimer. Upon activation of the PEB, the excitation energy is transferred to the central double-linked bilin584s, representing the delocalized excited state of coherence in the central dimer formation. The double-linked bilin584s create a highly electrically coupled excited state that facilitates rapid coherent energy transfer on the subpicosecond timescale. Furthermore, the lower-energy state of bilin618, which serves as the terminal acceptor for energy transfer, enables efficient energy transfer in PE566. These results reveal the internal EET mechanism of PE566 and provide insight into the diversity of photosynthetic systems in cryptophytes.

## Data Availability

The original contributions presented in the study are included in the article/**Supplementary Material**. Further inquiries can be directed to the corresponding author.
