# Supplementary material for: Ultrafast exciton energy transfer dynamics in the cryptophyte light-harvesting antenna phycoerythrin 566
Source: Front Plant Sci. 2025 Dec 2;16:1682154. doi: 10.3389/fpls.2025.1682154 (PMC12705538; doi:10.3389/fpls.2025.1682154)
Supplement: Supplementary file 1 [file DataSheet1.pdf]

Supplementary Material

1 Supplementary Figures

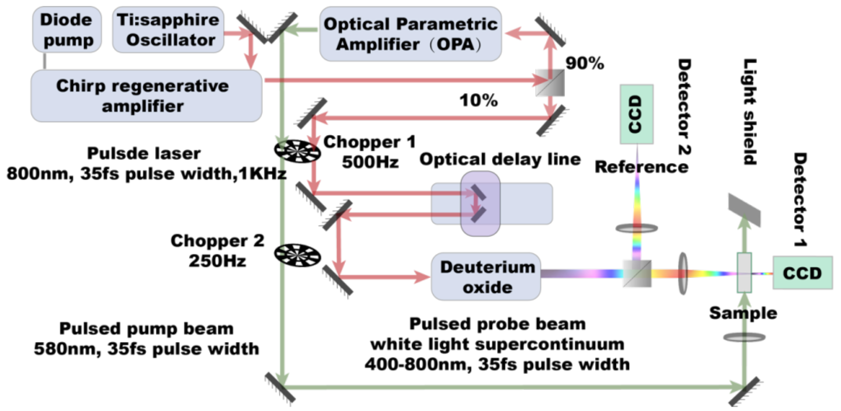

Figure S1. Schematic diagram of femtosecond transient absorption optics.

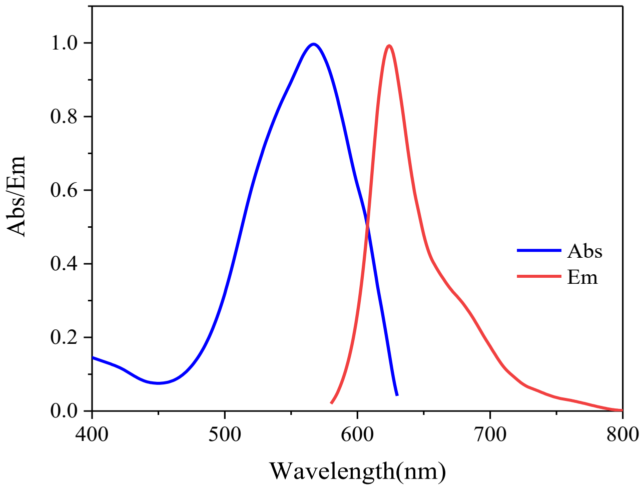

Figure S2. The absorption (blue line) and emission (red line) spectra of PE566

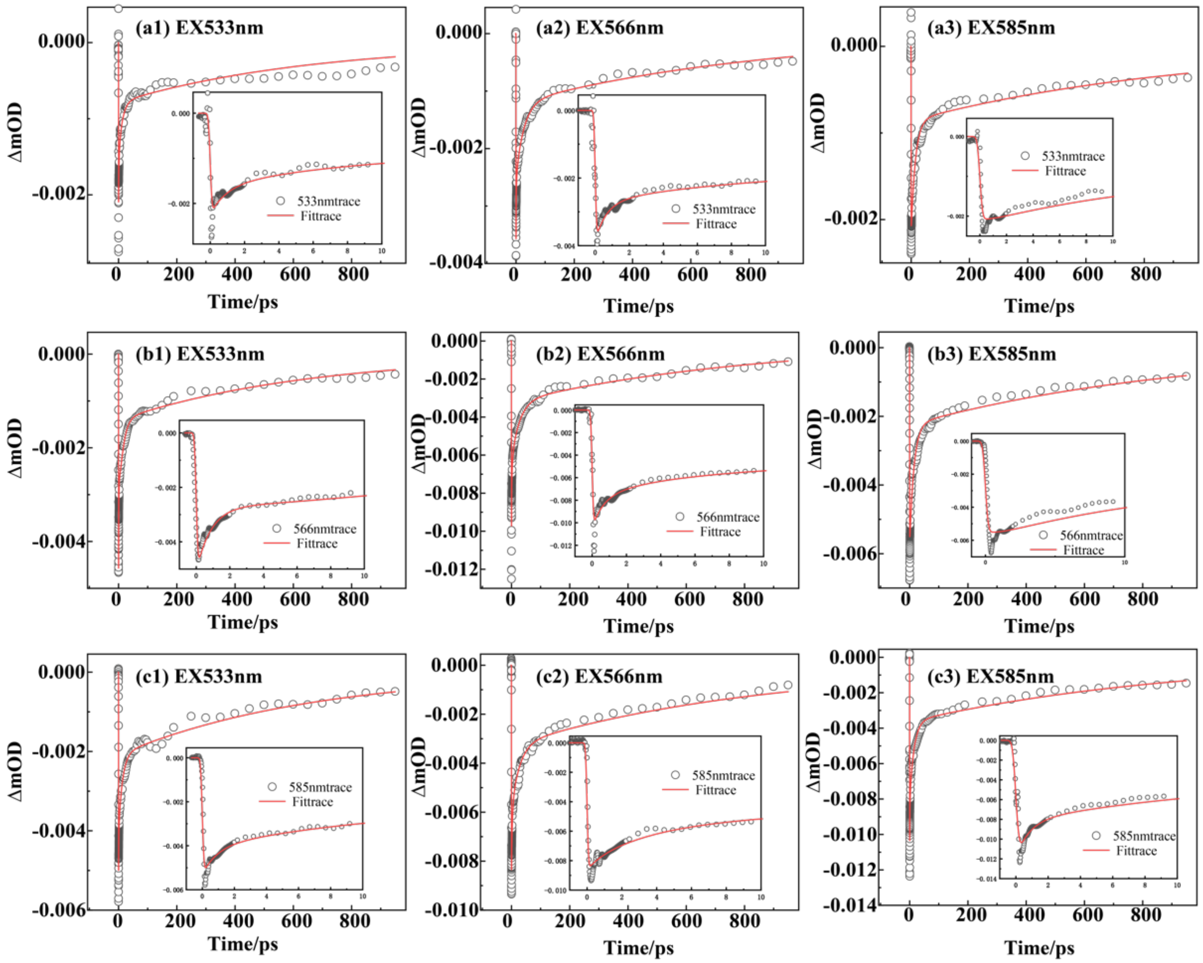

Figure S3. Characteristic wavelength kinetics of PE566. (a1),(a2),(a3) Excitation was done at 530nm. (b1),(b2),(b3) Excitation was done at 566nm. (c1),(c2),(c3) Excitation was done at 585nm. Squares represent the experiment data and red solid lines represent the single- or two-exponential fitting results.

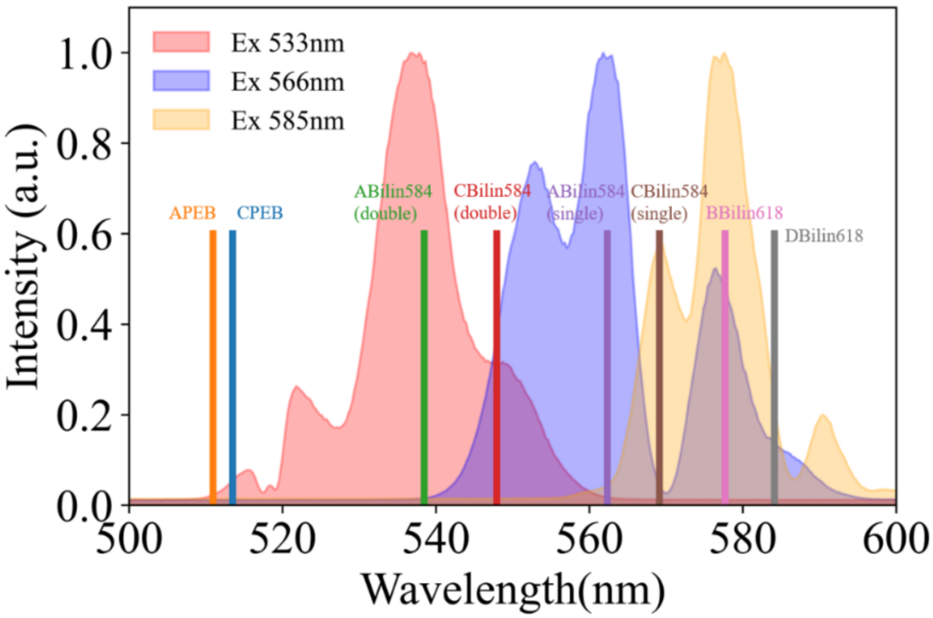

Figure S4. Pump pulse spectrum used in the experiment (filled) and excited state energy levels of the chromophores inside PE566 (solid line).

2 Supplementary calculation parameter

2.1 PE566 Hamiltonian (unit cm^{-1})

$$H_{PE566} = \begin{pmatrix} 19474.03 & -5.55 & 70.76 & 32.56 & -12.50 & -10.77 & 21.97 & 92.02 \\ -5.55 & 19571.94 & -29.35 & -70.83 & 12.01 & 13.93 & 86.52 & 21.60 \\ 70.76 & -29.35 & 18571.04 & -200.28 & 44.23 & -26.39 & -3.20 & 66.27 \\ 32.56 & -70.83 & -200.28 & 18250.83 & -29.71 & 52.78 & -73.81 & 3.63 \\ -12.50 & 12.01 & 44.23 & -29.71 & 17782.97 & -10.89 & -60.41 & -5.88 \\ -10.77 & 13.93 & -26.39 & 52.78 & -10.89 & 17570.84 & 6.34 & 64.48 \\ 21.97 & 86.52 & -3.20 & -73.81 & -60.41 & 6.34 & 17310.35 & -8.92 \\ 92.02 & 21.60 & 66.27 & 3.63 & -5.88 & 64.48 & -8.92 & 17120.00 \end{pmatrix}$$

2.2 The spectral density function and the recombination energy of each chromophore used in this paper

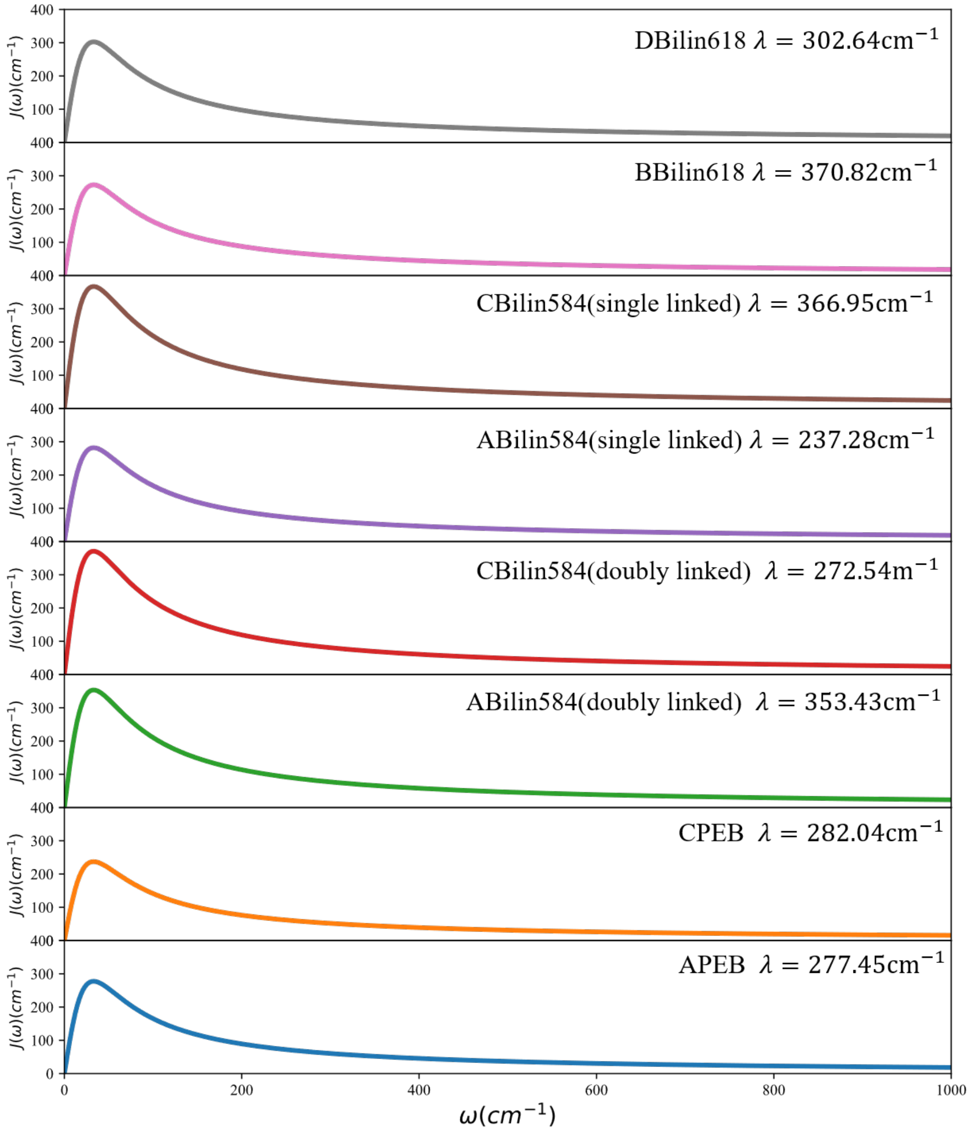

2.3 QM-oniom computational transition dipole moments of chromophores in PE566.

Chromophore	Transition dipole moments (Debye)			
	D_x	D_y	D_z	D_{tot}
APEB	2.90	0.38	2.71	3.99
CPEB	-3.40	-1.71	-0.79	3.89
ABilin584(double)	1.87	3.09	-0.15	3.62
CBilin584(double)	0.81	-2.37	3.50	4.30
ABilin584(single)	0.90	-2.27	-3.15	3.98
CBilin584(single)	0.23	-3.58	-1.46	3.87
BBilin618	1.34	3.33	-0.22	3.60
DBilin618	1.75	1.10	-3.70	4.24

2.4 The exciton state energy transfer rate $K_{kk'}$ under the CMRT framework

Table S1. Energy transfer rate between exciton states $|k\rangle$ and $|k'\rangle$, $K_{kk'}$ in ps^{-1}

Doner / Accepter	Ex1	Ex2	Ex3	Ex4	Ex5	Ex6	Ex7	Ex8
Ex1		2.758	0.486	0.094	0.005	0.003	0.024	0.189
Ex2	3.156		0.082	0.340	0.004	0.010	0.134	0.004
Ex3	2.506	0.216		21.54	0.363	0.235	0.072	0.140
Ex4	0.659	1.125	102.0		0.074	0.733	0.629	0.112
Ex5	0.031	0.006	1.618	0.204		0.355	1.900	0.029
Ex6	0.004	0.028	0.400	1.939	0.430		0.060	2.745
Ex7	0.032	0.223	0.196	3.644	5.714	0.067		0.459
Ex8	0.302	0.012	0.670	0.204	0.114	7.131	0.460	

3 Detailed derivation of EET dynamic

To further calculate the evolution of the system described in Eq. (5) in maintext, we employ projection operator techniques (Zwanzig, 1961; Mori, 1965),via

$$\mathcal{P}\rho = \rho_{ph} Tr_{ph}[\rho] \qquad (S1)$$

and

$$\mathcal{Q} = 1 - \mathcal{P} \qquad (S2)$$

By applying $\mathcal{P}$ to the density matrix in the interaction representation $\rho^I(t)$, and using the commutation of $\mathcal{P}$, the von Neumann equation becomes

$$\frac{d}{dt}\mathcal{P}\rho^I(t) = -i\mathcal{P}\mathcal{L}(t)\mathcal{Q}\rho^I(t) \qquad (S3)$$

$$\frac{d}{dt}\mathcal{Q}\rho^I(t) = -i\mathcal{L}(t)\mathcal{P}\rho^I(t) - i\mathcal{Q}\mathcal{L}(t)\mathcal{Q}\rho^I(t) \qquad (\mathrm{S4})$$

where $\mathcal{L}$ is the Liouville super operator, with $\mathcal{L}(t) = \left[H^{'}(t),\cdot\right]$, and

$$\begin{aligned}\rho^I(t) &= e^{iH_0 t}\rho(t)e^{-iH_0 t},\\ H^{'}(t) &= e^{iH_0 t}H^{'}e^{-iH_0 t}\end{aligned} \qquad (S5)$$

By expressing $\mathcal{Q}\rho^I(t)$ as a function of $\mathcal{P}\rho^I(t)$, and using the time-ordered exponentials, we have

$$\mathcal{Q}\rho^I(t) = -i\int_0^t d\tau\, \mathcal{L}(\tau)\mathcal{P}\rho^I(t) + \exp_+\left\{-i\int_0^t d\tau \mathcal{Q}\mathcal{L}(\tau)\right\}\mathcal{Q}\rho(0) \qquad (S6)$$

Here for a function $f(t)$, the time-ordered exponentials is defined as

$$\exp_+\left\{-i\int_0^t d\tau f(t)\right\} = \sum_{n=0}^{\infty}(-i)^n\int_0^t dt_1\int_0^{t_1} dt_2 \cdots \int_0^{t_{n-1}} dt_n f(t_1)f(t_2)\cdots f(t_n) \qquad (S7)$$

If the phonon is initially in the canonical distribution(Yang and Fleming, 2002; Trushechkin, 2019)， $\mathcal{Q}\rho(0) = 0$. Combining Eq. (8) with Eq. (11), and we have

$$\frac{d}{dt}\mathcal{P}\rho^I(t) = -\mathcal{P}\mathcal{L}(t)\int_0^t d\tau\, \mathcal{L}(\tau)\mathcal{P}\rho^I(\tau) \qquad (S8)$$

We employ the second Born approximation(Breuer and Petruccione, 2007), then we get the quantum master equation (QME) for the projected density matrix (the diagonal element of RDM in exciton basis):

$$\dot{\sigma}_{kk}(t) = \sum_{k^{'}\neq k}\left[K_{kk^{'}}(t)\sigma_{k^{'}k^{'}}(t) - K_{k^{'}k}\sigma_{kk}(t)\right] \qquad (S9)$$

Where $\sigma(t) = Tr_{ph}[\rho(t)]$, represents the RDM. And the transfer rates from $k^{'}$ -th exciton to k-th exciton $K_{kk^{'}}(t)$ is expressed as

$$K_{kk^{'}}(t) = 2Re\int_0^t d\tau\, Tr\left[e^{iH_{k^{'}k^{'}}\tau}H_{k^{'}k}^{'}e^{-iH_{kk}\tau}H_{kk^{'}}^{'}\rho_{k^{'}}\right] \qquad (S10)$$

Combining Eq. (15) with Eq. (5), and using the generating function equation(Yang and Fleming, 2002) and the cumulant expansion method(Silbey, 1996), we get the nonequilibrium, time-dependent energy transfer rate in exciton basis:

$$K_{kk^{'}}(t) = 2\mathrm{Re}\int_0^t d\tau F_{k^{'}}^{*}(\tau)A_k(\tau)N_{kk^{'}}(\tau) \qquad (S11)$$

REFERENCES

Breuer, H.-P., and Petruccione, F. (2007). *The theory of open quantum systems*., 1st Edn. Oxford University PressOxford. doi: 10.1093/acprof:oso/9780199213900.001.0001

Mori, H. (1965). Transport, collective motion, and brownian motion. *Prog. Theor. Phys.* 33, 423–455. doi: 10.1143/PTP.33.423

Silbey, R. J. (1996). Principles of nonlinear optical spectroscopy by shaul mukamel (university of rochester). Oxford university press: new york. 1995. xviii + 543 pp. $65.00. ISBN 0-19-509278-3. *J. Am. Chem. Soc.* 118, 12872–12872. doi: 10.1021/ja965513d

Trushechkin, A. (2019). Calculation of coherences in förster and modified redfield theories of excitation energy transfer. *J. Chem. Phys.* 151, 74101. doi: 10.1063/1.5100967

Yang, M., and Fleming, G. R. (2002). Influence of phonons on exciton transfer dynamics: comparison of the redfield, förster, and modified redfield equations. *Chem. Phys.* 275, 355–372. doi: 10.1016/S0301-0104(01)00540-7

Zwanzig, R. (1961). Memory effects in irreversible thermodynamics. *Phys. Rev.* 124, 983–992. doi: 10.1103/PhysRev.124.983
